# Butterfly: μW Level ULP Sensor Nodes with High Task Throughput

**DOI:** 10.3390/s22083082

**Published:** 2022-04-17

**Authors:** Chong Zhang, Li Lu, Yihang Song, Qianhe Meng, Junqin Zhang, Xiandong Shao, Guangyuan Zhang, Mengshu Hou

**Affiliations:** School of Computer Science and Engineering, University of Electronic Science and Technology of China, Qingshuihe Campus, Chengdu 611731, China; zhangchong@std.uestc.edu.cn (C.Z.); songyihang@std.uestc.edu.cn (Y.S.); qianhe@std.uestc.edu.cn (Q.M.); 202121081009@std.uestc.edu.cn (J.Z.); 202121081011@std.uestc.edu.cn (X.S.); 202122081013@std.uestc.edu.cn (G.Z.); mshou@uestc.edu.cn (M.H.)

**Keywords:** IoT, data efficiency, wireless sensing, ultra-low-power, security

## Abstract

The rapid development of Internet of Things (IoT) applications calls for light-weight IoT sensor nodes with both low-power consumption and excellent task execution efficiency. However, in the existing system framework, designers must make trade-offs between these two. In this paper, we propose an “edge-to-end integration” design paradigm, Butterfly, which assists sensor nodes to perform sensing tasks more efficiently with lower power consumption through their (high-performance) network infrastructures (i.e., a gateway). On the one hand, to optimize the power consumption, Butterfly offloads the energy-intensive computational tasks from the nodes to the gateway with only microwatt-level power budget, thereby eliminating the power-consuming Microcontroller (MCU) from the node. On the other hand, we address three issues facing the optimization of task execution efficiency. To start with, we buffer the frequently used instructions and data to minimize the volume of data transmitted on the downlink. Furthermore, based on our investigation on typical sensing data structures, we present a novel last-bit transmission and packaging mechanism to reduce the data amount on the uplink. Finally, we design a task prediction mechanism on the gateway to support efficient scheduling of concurrent tasks on multiple MCU-free Butterfly nodes. The experiment results show that Butterfly can speed up the task rate by 4.91 times and reduce the power consumption of each node by 94.3%, compared to the benchmarks. In addition, Butterfly nodes have natural security advantages (e.g., anti-capture) as they offload the control function with all application information up to the gateway.

## 1. Introduction

Ultra-Low-Power (ULP) consumption and high task efficiency are two urgently needed for Internet of Things (IoT) sensor nodes. In the current architecture, however, the task performance of IoT sensor nodes is tightly coupled with its power consumption, and thus most of the IoT applications have to make trade-offs between them. The reason is that, on existing sensor nodes, all the tasks are executed using a general-purpose MCU, and thus the optimization for power consumption is essentially realized at the expense of high MCU working speed.

In state-of-the-art studies, a rich set of methods for low-power sensor nodes design are proposed in two ways. One branch focuses on switching the working mode of the MCU, aiming to reduce average power overhead of the node by limiting the high-speed operating of MCU. For instance, ULP sensor nodes like WISP [1], MOO [2] and LILAC [3] suffer from MCU working power at the milliwatt-level (mWs) and have to lower their duty cycle to save energy [4,5]. However, the low duty cycle inversely limits the task execution efficiency. Another direction is proposed based on edge computing [6,7,8] where heavy tasks can be offloaded from sensor nodes to a high-performance gateway, thereby optimizing the power and task efficiency of the nodes. However, making offloading decisions requires the MCU as well as the programs running on it, which brings extra computing and power overhead, and thus becomes the bottleneck of further reducing the power consumption.

The fundamental cause for these aforementioned issues is that the existing optimization efforts are presented for the MCU-based sensor node architecture, which always needs to run embedded programs with either high power or low duty cycle. With this fact in our mind, in this paper, we ask if we can introduce a new architecture that can achieve high task efficiency without incurring the high power of MCU. To this end, we should address two basic issues, i.e., (1) how to efficiently realize sensing function without involving the MCU, and (2) how to achieve high task efficiency on such a dumb MCU-free sensor node.

In this paper, we propose Butterfly, a methodology for the design of lightweight sensor nodes with both ULP overhead and high task efficiency. Our basic idea is to design a μW level digital circuit with Finite State Machine (FSM) functions to replace the general MCU for sensors control and offload all remaining tasks to an edge gateway via ULP communications. Then, by designing an efficient data transmission and utilization mechanisms in the FSM, Butterfly can minimize the volume of data required by computing offloading, and thus minimize the communication time cost to support rapid task execution on μW level ULP Lightweight IoT sensor nodes (LSN)s. However, to turn our butterfly design into a practical working system, we need to address the following three technical obstacles that need to be settled urgently.

First, it is non-trivial for an LSN to read sensors efficiently without a general MCU. Specifically, most digital sensors utilize computer bus interfaces (In the initial state, we consider controlling digital sensors via digital bus interfaces. For analog sensors, an analog-to-digital conversion unit can be added to the circuit in practical applications.) for interactions, which were once accessed by the MCU with running embedded programs. However, as the MCU is removed for ULP design, it is hard for an LSN to provide required bus signals to access on-board sensors efficiently. Specifically, the possible approach might be to utilize the gateway to directly control sensors on LSNs via wireless signals, where the LSN converts the received wireless signal into bus signals for sensor control. However, a complete bus interface contains multiple signal lines with strict timing. Transmitting the full information of bus signals will bring a huge load on wireless communication and ultimately become obstacles to the efficient sensor reading. To settle this issue, we designed an Efficient Bus Signal Transmission (EBST, Section 4) mechanism to convert wireless gateway signals into Serial Peripheral Interface (SPI) bus signals for sensor control with high efficiency. Compared with the previous work [9], this mechanism achieves four times higher transmission efficiency.

Second, it is hard for an LSN to achieve rapid task execution under a given communication data rate with frequent gateway interactions for IoT applications. Specifically, since application functions and sensor control are separately operated on the gateway and LSN, it requires frequent interactions between them with diverse data formats according to various IoT applications. However, the LSN has a limited data rate due to the limited power budget, which inevitably obstructs the rapid execution of sensing tasks. To solve this challenge, we design a Data-Efficient Sensor Control (DESC, Section 5) mechanism with simple FSM logic to cut down communication overhead on both downlink and uplink aspects. By buffering frequently used commands and instructions in downlink and removing sensory data redundancy in the uplink, an LSN can perform mass sensing tasks with minimized time cost on data transmission, thus significantly accelerating the task rate.

Third, it is difficult for the edge gateway to manage tasks on multiple LSNs when they need to be operated in parallel. It may lead to possible task failures caused by multiple LSNs that upload sensory data simultaneously, which cause data collisions. To settle this issue, we design a task prediction mechanism on the edge gateway, which calculates future bandwidth occupation for each LSN based on its running mode, clock frequency and recorded time mark in previous data uploading. By this, the edge gateway can effectively predict the time available for future communication resources to better manage parallel tasks on multiple LSNs and further improve efficiency. The details are presented in Section 6.

We implement a Butterfly prototype system for the proof of concept, i.e., multiple Butterfly LSNs based on ULP FPGA (IGLOO nano-AGLN060) and an edge gateway based on software-defined radio (USRP-2922). We conduct extensive field experiments and simulations with the prototype system. The results demonstrate that a Butterfly LSN can sample a 12-bit ADC at 137 kHz with only 703 μW consumption, which only needs 5 nJ power for each sample. In addition, compared with traditional ULP sensor nodes, the LSN can accelerate 4.91 times the maximum task rate and reduces the power consumption by 94.3%, respectively. In addition, as all application information is shifted to execute on the gateway, the LSN achieves anonymity security, as it contains no private information, thus anti-capture for attackers. The main contributions made in this work are as follows:

- We propose a new methodology for the design of lightweight IoT sensor nodes, which has μW level power consumption, rapid task performance and anonymity security.
- We design an efficient bus signal transmission mechanism for sensor control on MCU-free LSNs, which achieves four times the efficiency higher than the previous work.
- We design a data-efficient sensor control mechanism, which removes data redundancy in both downlink and uplink aspects, and thus further accelerates the task rate.
- We design a task prediction mechanism to manage sensing tasks on multiple LSNs, which can significantly improve the execution efficiency of concurrent tasks.

The rest of this paper is organized as follows: in Section 2, we introduce state-of-the-art related works and provide a background for our design. In Section 3, we present our system overview. Next, in Section 4, we introduce the detailed design towards efficient signal transmission, which corresponds to the first challenge in the introduction. Then, in Section 5 and Section 6, we present the design of data-efficient sensor control and task prediction mechanisms, which correspond to the second and third challenges in the introduction, respectively. Furthermore, the implementation and evaluation of Butterfly are illustrated in Section 7 and Section 8, respectively. Next, we discuss security and other concerns in Section 9. Finally, we conclude this paper in Section 10.

## 2. Related Works 

Ultra-low-power consumption and excellent task performance are two coveted indicators that are pursued by IoT sensor nodes, where the former enables the node to achieve longer battery life, prolong maintenance cycles and can thus significantly reduce application costs under large-scale needs. In addition, the latter gives the nodes a better versatility to meet the requirements of diverse applications. Under the needs of large-scale applications, a rich set of existing schemes have been proposed, aiming to reduce the power consumption of IoT sensor nodes, however, all of which are achieved at the expense of device performance. For a better presentation, we present the state-of-the-art related works in two ways as follows:

(1) Work mode optimized on MCU. This branch focuses on optimizing the power consumption of the node by adjusting the operating mode of its MCU, such as WISP [10], MOO [2], LILAC [3] and many other battery-less nodes [11,12,13] which can even work with ambient power [14,15,16,17,18]. For these works, the indispensable design lies in the operating state control of MCU, where methods of duty-cycle [19] and DVFS [20] are useful to reduce its average power consumption by switching the MCU’s operating state or clock frequency. However, since the total workloads for the same tasks remain unchanged, such approaches hardly cut down the total power consumption in the task execution but may even bring additional energy overhead due to frequently switching the working states of MCU. Finally, the above approaches principally reduce the average running speed of MCU, which eventually obstructs the node to achieve a good task efficiency.
(2) Computing offloading. This direction focuses on computing offloading that utilizes gateways to share part of the computing tasks with the node and optimize its power consumption. There are representative works like literature [21,22,23,24,25], where many useful methods are proposed to share tasks with sensor nodes and the edge gateway for lower power overhead. However, as the decision of offloaded tasks requires additional program functionality in the MCU, it brings extra computing and communication loads and thus becomes the bottleneck of further reducing the power consumption.

**Figure 1:**
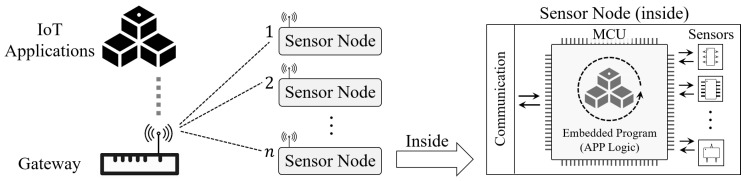
A general operating architecture of IoT sensor nodes, where each node works as an autonomous subsystem that functioned by an on-board MCU running embedded programs, i.e., read sensors and upload the collected sensory data. The gateway works as a data relay that collects the uploaded sensory data from distributed nodes and provides them for corresponding IoT applications.

The major barrier that obstructs the above works to achieve both ULP design and high task efficiency is the MCU-based architecture, where each IoT sensor node is generally operated as an autonomous and independent system, as shown in the Figure 1. The MCU runs a built-in embedded program for sensor control to control the node for sensing and data collection. In recent years, benefits by ULP sensors [26,27] and ULP communications (e.g., backscatter [28,29,30,31]), the power overhead of sensing, and communication can drop to the μW scale. However, the MCU, which is in essence a microcomputer in Harvard or Von Neumann architecture [32] that executes all operations by a complex series of steps (e.g., fetching, decoding, addressing), becomes the final obstacle for ULP sensor nodes to further optimize power overheads. In principle, all power optimizations on MCUs are achieved at the expense of their running speed, which inevitably sacrifices the task performance of IoT sensor nodes. For instance, in [33], the authors adopt a duty-cycle scheme on an MSP 430 MCU to help the node operate under the RF power supply. However, to satisfy the ULP requirements, the MCU splits a simple CRC16 calculation into 16 parts and takes more than 6 s to complete (which only needs 840 μS originally).

To settle the high power issue of MCU, recently, the Radio-to-Bus (R2B) [9] designed a set of circuits to convert RF signals into SPI Bus signals for sensor control directly via a gateway. By this, general MCU can be removed from the node and achieves 4.5 times lower power overhead than traditional works. However, to achieve sensor control without the help of a general MCU, it needs to convey the complete information of bus signals, thus bringing huge time costs on wireless transmission and eventually limiting the task efficiency. Specifically, this work adopts a PIE encoded signal to convey both the data and clock information of the SPI bus in a symbol. Considering the maxim processable frequency of wireless receiver on the node is *f*, then the shortest interval between logic high and logic low transitions could be defined as τ ($\tau =\frac{1}{f}$). In this work, it requires 5 τ to transmit the symbol of data 1 (4 τ for logic high and 1 τ for logic low) and 3 τ to transmit the symbol of data 0 (2 τ for logic high and 1 τ for logic low). Hence, the achievable data rate is only $\frac{1}{4}f$ (25% of the circuit process capabilities). In addition, to handle sensor control in multi nodes scenarios, mass extra information is required to be transmitted for system control, e.g., chip select, node ID, etc., which further reduce the transmission efficiency. As a result, to transmit 1 bit of valid bus data to the sensor, it usually takes 10 to 20 τ in the time cost.

In summary, to the best of our knowledge, there is no successful solution that achieves ULP designs on IoT sensor nodes without sacrificing the task execution efficiency. To achieve both ULP design and high task efficiency simultaneously, it is necessary to design a μW scale logic circuit for sensor control that replaces the general MCU and is also vital to building up efficient data transfer and utilization mechanisms that cut down communication overhead. The design should effectively avoid excessive dependencies with the gateway without the help of a general MCU.

## 3. Butterfly in a Nutshell 

**Figure 2:**
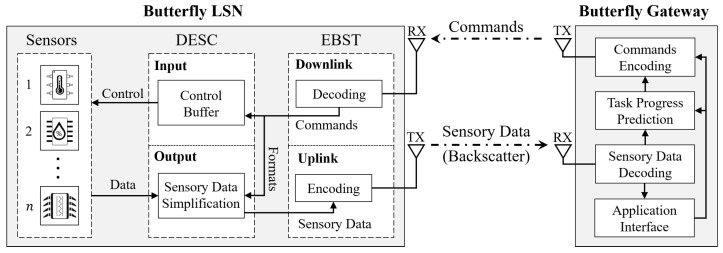
The architecture of a Butterfly system, which consists of a gateway and distributed LSNs connected via a wireless network.

The core idea of Butterfly design is to offload all task logic from distributed LSNs to a centralized gateway and remove data redundancy in the communication links. By this, both computing and communication overhead on LSNs can be significantly reduced, which minimizes its power consumption while keeping good task efficiency. As depicted in Figure 2, a Butterfly system is typically constituted by a number of Butterfly LSNs and an edge gateway.

### 3.1. Butterfly LSN

A Butterfly LSN works as an interpreter that connects the gateway with its deployed sensors, which consists of the following three units:

(1) Efficient Bus Signal Transmission (EBST): For a Butterfly LSN to execute tasks rapidly, the EBST unit plays an essential role, handling efficient signal converting in both downlink and uplink aspects. Specifically, on the downlink side, the EBST decodes the received wireless signals in an efficient way and converts it into standard computer bus signals, which the LSN and deployed sensors can recognize. In addition, in the uplink aspects, the sensory data are conveyed to the gateway via backscatter with ultra-low-power consumption. With efficient signal converting, the EBST unit gives an essential foundation for the LSN to control sensors and execute sensing tasks rapidly. Compared with the previous work [9], our EBST theoretically improves 400% efficiency in downlink bus signal transmission (as described in Section 4.1).
(2) Data-Efficient Sensor Control (DESC): The DESC unit is the key element that further accelerates the task rate of sensor reading for LSNs. By buffering the frequently used commands in input aspects and simplifying the sensory data by its formats in output aspects, a DESC can minimize the amounts of transmitted data between the gateway and LSNs in both downlink and uplink aspects. By this, even a small amount of data transmission can support the execution of a large number of tasks, thereby further improving task performance and efficiency. The detailed design is presented in Section 5.
(3) Onboard sensors: The Commercial Off-The-Shelf (COTS) sensor can be directly purchased online or offline. We connect the sensor to our LSN for feasibility verification and performance evaluation.

### 3.2. Butterfly Gateway

The gateway works as a master that coordinates the access to all sensors deployed on one or multiple LSNs in the system, which consists of the following four components:

(1) Commands Encoding unit: This unit encodes downlink commands for the LSN control. According to the application requirements and current task status, this unit issues corresponding commands to control the target LSN to perform operations such as buffer writing, sensor reading, etc.
(2) Task Progress Prediction (TPP): The TPP unit is a key component that is designed to predict the task execution states of each LSN to better utilize resources in a gateway network and further improve the task efficiency. Specifically, based on the running speed of each LSN, this unit can calculate the possible timing for future bandwidth occupation, which also indicates the available part that the gateway can issue commands to execute other tasks simultaneously. Such a design can effectively reduce the task delay and cut down the waiting time for sensory data receiving, thereby effectively improving the execution efficiency of concurrent tasks. In addition, it can efficiently avoid possible data collisions caused by multi-LSNs uploading the data simultaneously. The detailed designs are discussed in Section 6.
(3) Sensory data decoding: This unit is designed to decode the uploaded sensory data and provide it for corresponding IoT applications through the application interface. At the same time, the decode results are also provided to the task prediction unit to obtain current task progress of the target LSN.
(4) Application interface: The interface for IoT users to enter the requirements for specific IoT applications, e.g., reading an accelerometer at 1.2 kHz for 10 s on a specific LSN. Based on the recorded requirements, the gateway controls the target LSN to execute corresponding sensing tasks accordingly.

In the following three sections (Section 4, Section 5 and Section 6), we discuss the design of the three key units: Efficient Bus Signal Transmission (EBST), Data-Efficient Sensor Control (DESC), and Task Progress Prediction (TPP) in detail, which correspond to the three challenges we mentioned in the Introduction (Section 1).

## 4. Efficient Bus Signal Transmission (EBST)

Efficient signal transmission is the basis for rapid task execution. As most digital sensors can be accessed by computer bus signals (e.g., I2C, SPI), an efficient RF signal to bus signal conversion and transmission mechanism is required to be designed on the LSN to replace the MCU for efficient interactions with on-board sensors, which transmit control commands and data for the target sensor in the downlink and return sensory data to the gateway in the uplink.

At the initial state, we take the transmission of the SPI bus signal as an example, and the design in downlink (bus signal input) and uplink (bus signal output) aspects are presented as follows.

### 4.1. Downlink Signal Transmission 

An ordinary SPI Bus interface mainly contains four kinds of lines, i.e., a signal line for Serial Data Input (SDI), a signal line for Serial Data Output (SDO), a signal line for synchronous clock input (SCK) and one or multiple signal lines for chip select (CS) input (each deployed sensor needs a CS line for chip select control). In downlink transmission (signal input), at least three kinds of signals need to be transmitted (SDI, CLK and CS), which would bring a huge load in wireless communication and ultimately sacrifice task execution efficiency (as described in Section 2). Hence, we consider only transmitting the information on the data input line in the SPI Bus interface and generating the synchronous clock locally on the LSN. For the CS signal, it can be generated via instructions in digit circuit (the details are present in Section 5). The detailed design is presented in the following:

In the data line, data ‘1’ and data ‘0’ are represented by logic high and logic low, respectively. In addition, the synchronous clock is designed for the sensor to receive the data correctly, where each bit of the data are recognized at the rising edge (mode 0) or falling edge (mode 1) of the clock [34]. To generate SPI signals efficiently, we designed a circuit to decode the received RF signal, where each τ of wireless signal transmission can generate one bit of bus data (considering that the shortest duration of logic high and logic low in the baseband signal is τ. In the corresponding, the maximum signal changing frequency that the LSN needs to handle is $\frac{1}{\tau }$).

**Figure 3:**
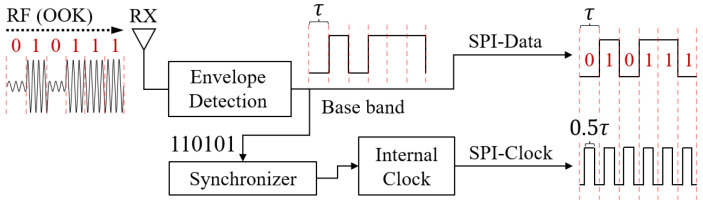
In the downlink, the LSN efficiently converts gateway wireless signals into on-board SPI bus signals for sensor access.

As illustrated in Figure 3, in the downlink aspect, the received RF signals are first decoded into baseband signals by a passive envelope detection circuit with ULP design (we utilize the same circuit like [9], which works in few μWs). In order to improve the transmission efficiency, we directly utilize the decoded baseband signal as the data line of the SPI bus. In this way, the gateway only needs to modulate the signal of SPI data on the carrier through Amplitude Shift Keying (ASK), and the LSN can directly get the corresponding signal after envelope detection, thus achieving the 1 to 1 transmission efficiency from wireless signal to bus data.

Next, to generate the synchronous clock, we set a local internal clock that has the same period (τ) with the downlink wireless signal. In order to keep the synchronization, we design a synchronization frame (110101) and a synchronizer. Specifically, the synchronization frame will be transmitted at the beginning of each downlink packet. In addition, in a large data package, the synchronization frame will be sent again after every (*N*) bit of data transmission to avoid possible data error caused by frequency deviation. As a result, we obtain the SPI data and clock signals as

(1) $$f_{data}\left(t\right)=\left\{\begin{array}{c} H_{level},0\leq t<\tau \;\;\;(Data\;\;1)\\ L_{level},0\leq t<\tau \;\;\;(Data\;\;0) \end{array}\right.$$

(2) $$f_{clock}\left(t\right)=\left\{\begin{array}{c} H_{level},\frac{1}{4}\tau \leq t<\frac{3}{4}\tau \\ L_{level},0⩽t<\frac{1}{4}\tau \&\frac{3}{4}\tau ⩽t<\tau \end{array}\right.$$

where $H_{level}$ and $L_{level}$ denote the logic-high and logic-low voltages, respectively; $f_{data}$ and $f_{clock}$ denote the data and clock signals in the SPI bus, respectively.

Based on the above design, we have obtained the 1:1 transmission efficiency of wireless data to bus data, which is 400% more efficient than the previous work [9]. Since the minimum change interval (τ) between the logic high and logic low in the baseband remains the same, which brings the same processing load for the envelope detection circuit, thus requiring no need for circuit parameter modification and avoiding the increase of power consumption to the rapid rise of transmission rate. As a result, compared to the previous work, we have theoretically reduced the time cost by 75% for each bit of bus signal transmission by using the same envelope detection circuit.

Finally, in order to further improve the task efficiency, the generated bus signal will not be directly used for sensor control but first sent to the state machine for management, which buffers the common data and removes data redundancy to minimize the required amount of data transmission in the wireless channel. Such progress is discussed in detail in Section 5.

### 4.2. Uplink Signal Transmission

**Figure 4:**
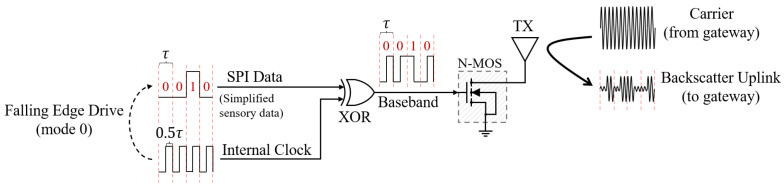
In the uplink aspects, the LSN uploads the de-redundant sensory data to the gateway via ultra-low-power backscatter communication.

In the uplink phase, we employ the backscatter communication [28] to convey the uploaded bus data to the gateway with ultra-low-power consumption. As illustrated in Figure 4, we utilize SPI mode 0 to output the de-redundant (as described Section 5) sensory data for uplink, i.e., the output of each bit of data is driven by the falling edge of the clock. To prevent the backscattered uplink signal from being swamped by the gateway carrier with much higher amplitude and power, we mix the bus data signal with the clock signal to increase the baseband frequency so that the backscattered signal can be shifted away from the carrier in frequency. The baseband signal changes the antenna impedance by controlling the on/off of a N-type MOSFET (N-MOS) switch, thus modulating the signal on the carrier provided by the gateway. Considering that the carrier frequency is $f_{0}$, the clock frequency is $f_{c}$, and the signal change frequency on the data line is $f_{d}$, the uplink backscatter frequency $f_{b}$ can be expressed as:

(3) $$f_{b}=f_{0}+f_{c}+f_{d}$$

## 5. Data-Efficient Sensor Control (DESC) 

One major barrier that obstructs the MCU-free ULP sensor nodes from executing tasks efficiently is the requirement of mass data transmission. Specifically, as the MCU is removed for ULP consumption, it causes heavy reliance on the gateway as the node cannot run embedded programs and control on-board sensors locally. In the literature [9], each step of sensor operations can only be achieved by issuing the gateway commands and thus brings huge communication overhead. For instance, if the reading of a deployed sensor contains operations like Chip Select (CS), read command input, read register address input and sensor data readout, as each operation is required to be executed by a wireless command with 16-bit length, it causes 64 bits of data transmission for a single sensor reading operation. At the same time, the sensory data uplink also needs to be accompanied by a large number of protocol transmissions (e.g., Preamble, device ID, etc.). Ultimately, although the design of the MPU-Less node brings advantages in ULP overhead, the huge overhead in both uplink and downlink transmission also causes obstacles for the rapid execution of sensing tasks. In order to avoid such a bad situation, we design a Data-Efficient Sensor Control (DESC) scheme in both the input and output sides for sensor interaction, buffering frequently used commands and removing redundancy in the sensory data. The detailed design is presented as follows:

### 5.1. Control Input Buffering

**Figure 5:**
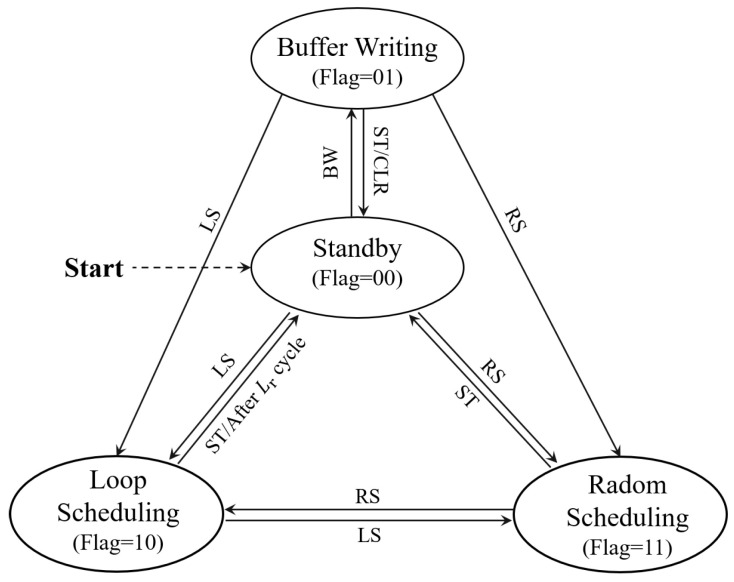
The main states and transfer logic of a Butterfly LSN.

**Figure 6:**
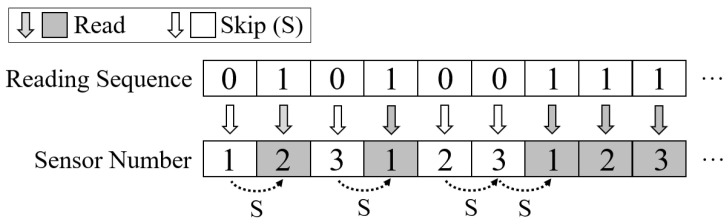
In the random scheduling mode, each bit of the scheduling sequence can determine the read or skip the operation for a selected sensor (0 for skip, 1 for read), thus significantly reducing communication overhead and improving task efficiency.

We observe that the control logic is fixed for the same sensor in general. Hence, there is no need to issue the commands with the full control sequence for the same sensor in every sampling. To this end, we designed a state machine logic to achieve efficient sensor control by buffering the control logic for every onboard sensor, as shown in Figure 5. The designed four working states in the FSM are as follows:

- Standby (flag = 00). When the LSN is powered on, it initially goes to the stand by state for commands receiving and will jump to another three states if the corresponding command is received. In addition, the gateway can also control an LSN from other states to jump to the standby state by issuing the standby (ST) command.
- Buffer Writing (flag = 01). The buffer writing state is a necessary state before a LSN performs sensor control, which is designed to buffer the frequent used information and thus save bandwidth in future operations. Specifically, in this state, the gateway can write the control sequence of a sensor into the buffer of the target LSN, with which all required underlying signal (including the required input signal in SPI interface) can be generated for sensor control. In addition, the configuration of sensory data de-redundancy in the uplink channel is also written by the gateway in this state. By issuing a buffer writing (BW) command, a gateway can transfer the target LSN into the buffer writing state; after that, the corresponding sensor can be operated with minimized communication overhead.
- Loop Scheduling (flag = 10). In this state, the LSN cyclically reads with one or more sensors selected, minimizing communication overhead. A Loop Scheduling (LS) command with loop configurations can transfer an LSN into this state, where the target sensors are sampled under the loop configuration—for instance, if the configuration represents reading three selected sensors for 500 cycles at 25 Hz. When received, the LSN can automatically read corresponding sensors for 500 times at the defined speed and upload the results after de-redundancy (as described in Section 5.2). For the gateway, it receives the uploaded sensory data and calculates the time mark of each sample based on the preset sample rate to recover the complete information of all sensory data. In this way, a large amount of complete sensor data can be obtained with minimized communication overhead. In addition, if the application needs, the gateway can issue an ST command or random scheduling (RS) command to interrupt the sensor reading and turn the LSN state into standby or random scheduling, respectively.
- Random Scheduling (flag = 11). In this state, the gateway can control sensors on the target LSN in a random sequence with minimized overhead. Specifically, in this state, each bit of the gateway command will correspond to the scheduling decision of a sensor, where 1 means read the current sensor and 0 means skip for the next sensor. For a better understanding, we make an example in Figure 6. For instance, if the gateway sends 010100111 to an LSN with three sensors selected to operate, it means read the 2-nd sensor in the first loop, read the 1-st sensor in the second loop, and read all sensors in the third loop. By this, each sensor can be decided to read or skip with only 1-bit of command transmission, ensuring a random order of sensor reading for the requirements of various IoT applications while minimizing communication overhead.

Based on the above design, the downlink data overhead can be greatly reduced for various IoT applications, thus achieving data-efficient task execution with ultra-low power consumption. In addition, for security consideration, the buffered data are only related to the control of Commercial Off-The-Shelf (COTS) sensors, which are public and do not contain any application logic and sensitive information. Hence, the LSN has natural security advantages (e.g., anti-capture) as all application-related information is offloaded to the gateway and the locally stored data are useless for attackers.

### 5.2. Sensor Output Simplification

The design of sensor output simplification mechanism consists of two parts, i.e., sensory data de-redundancy and data packaging, as follows:

#### 5.2.1. Sensory Data De-Redundancy

We observed that the sensory data output contains a lot of redundancy where most data bits will remain unchanged in two consecutive outputs from the same sensor. Specifically, the outputted sensory data have a certain functional relationship with the physical quantity as the sensor monitors, and the output data change a little if the change of the monitored physical quantity is far from the sensing range. In addition, many sensor outputs are accompanied by fixed data padding, which also increases the transferred data in total. For instance, TMP125 [35] is a 10-bit temperature sensor (TMP125), but its output is 16 bits in total, with only the 6-th to 15-th bits effective. In addition, if the sensed ambient temperature is 6${}^{\circ }$ and 15${}^{\circ }$ in the adjacent sample, its output will be 0000-0011-0000-0000 and 0000-0111-1000-0000, respectively. Hence, an effective method to improve the transmission efficiency is only transferring the four changed bits instead of all 16 bits. In practice, in scenarios with higher sampling frequency for sensors on a LSN, the variation in continuously sensory data could be minor. Hence, removing redundancy in sensory data could be an effective method to cut down the uplink overhead.

**Figure 7:**
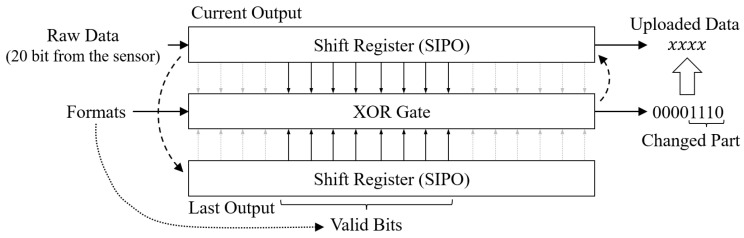
In the last bit transmission mechanism design, an XOR gate is deployed to compare variation between the adjacent outputted sensory data in two SIPO registers, which extracts the changed part of the sensory data for uploading to minimize the load in the uplink aspect.

To this end, we designed a last-bit transmission mechanism. By comparing the variation of the valid database on the data format of the sensor and only uploading the changed bits, an LSN can significantly reduce the uplink overheads and greatly improve the task efficiency in a given bandwidth, as shown in Figure 7.

We utilize two Serial-In, Parallel-Out (SIPO) shift registers to store the raw data from a sensor for the current output and the last output, respectively, and compare the Parallel outputted data bit by bit via an XOR gate. Based on the record format information (as described in Section 3.1), the XOR gate compares the valid bit of the sensory data and outputs the results for further processing. Specifically, the changed and unchanged bits are marked as 1 and 0, respectively. For instance, the XOR output (00001110) means the valid data are 8 bits at length, and the changed bits are the 2nd to 4th bits.

For the design purpose, we only want to transmit the three changed bits to minimize communication load in the uplink. However, to make the gateway recognize the uploaded data and recover the complete value, information about the location of changed bits is required, which in turn leads to the surge of communication overhead. Therefore, we consider transmitting all the bits from the first change to the end of valid data. With a fixed rule, there is no need to transmit the bit count information. Although some extra bits will be transmitted, it is much less than the bit count information or the raw sensory data. For instance, in the example in the figure, we need to transmit the last four bits of sensory data, which is one bit more than the changed bits but much less than the raw 20-bit data.

It should be noted that each sensor needs a set of such mechanisms, which do not introduce significant power overhead as the design is simple. In addition, in practice, few sensor deployments are often sufficient for one LSN for environment sensing.

#### 5.2.2. Sensory Data Packaging

**Figure 8:**
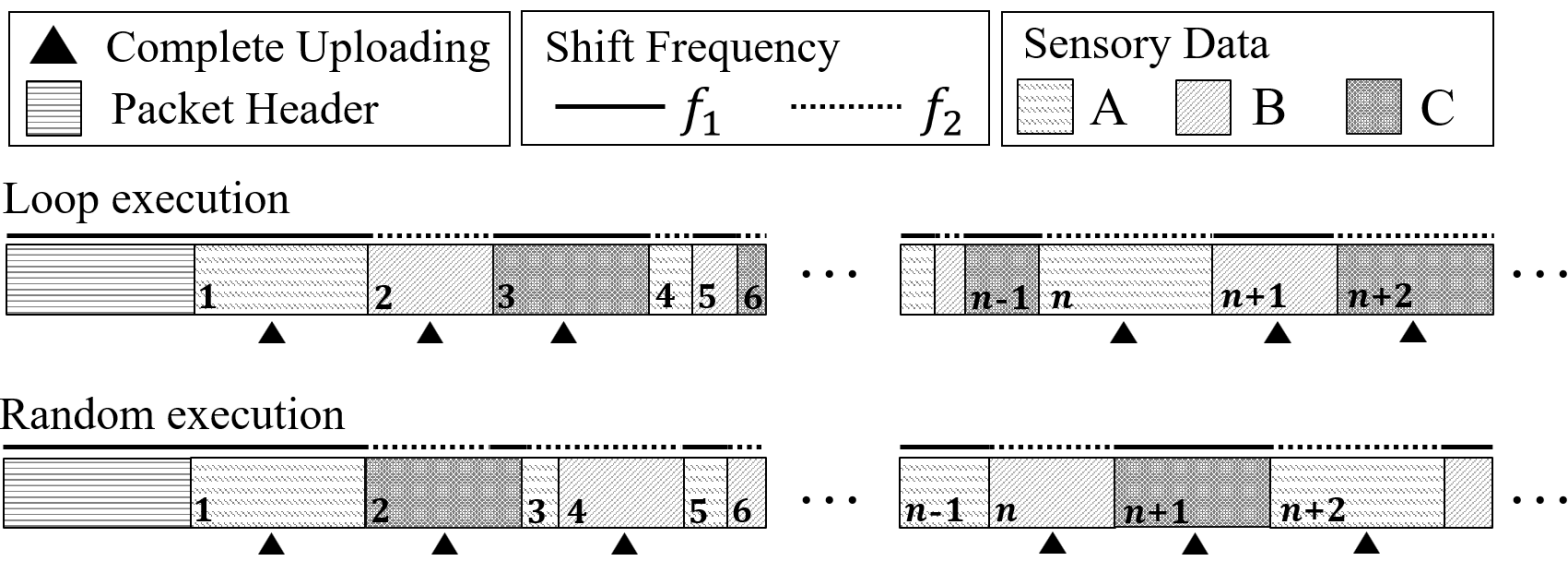
By packaging the simplified sensory data, a Butterfly LSN can effectively avoid fragmented usage in the uplink channel and significantly reduce the protocol overhead.

To avoid the fragmented of bandwidth usage, we combine the simplified sensory data in one package for uploading. It can also effectively reduce the protocol overhead as each packet needs to have a packet header that carries the leading code, device ID, length setting, etc. The design is presented in Figure 8.

Considering the simplified sensory data have random lengths, we utilize two different frequencies to transmit the adjacent sensory data. By this, the gateway can split the data from each sensor and recover the complete data for the corresponding IoT applications. In addition, with information on data transmission rates and sensor sampling rate of the target LSN, the gateway can precisely calculate the accurate time mark for each sensory data, thus recovering the completed trend of the corresponding monitored physical quantities. To enhance the robustness of the system, LSN performs a complete transmission for all sampled sensors in every *n* packet to avoid possible cumulative errors caused by communication bit errors. Finally, the length of the package can be set by the real-time requirements of IoT applications. In exceptional cases, each piece of sensory data can be uploaded separately to achieve high real-time sensing.

## 6. Butterfly Networks

**Figure 9:**
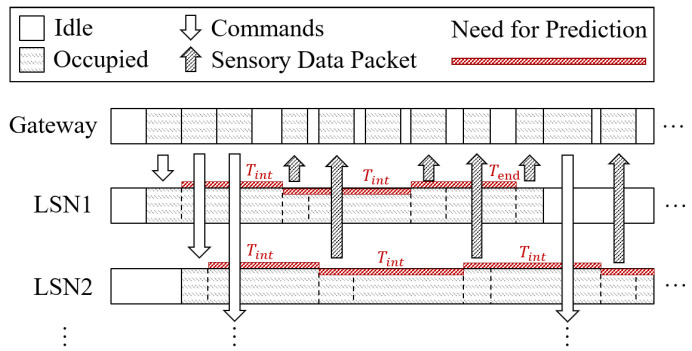
The edge gateway predicts the task execution progress on each LSN to estimate the possible bandwidth occupation in the future and optimize the time mark for each command issue. By this, the gateway can effectively extend the network capacity and support more LSNs in its control.

In a Butterfly network, the edge gateway issues downlink commands to manage tasks on multiple LSNs and predict when these LSNs may upload data, thus calculating future bandwidth availability for the issue of the next command, thereby improving bandwidth utilization and extending the network capacity of the gateway to support more LSNs, as shown in Figure 9. For a better understanding, we consider discussing the task perdition of LSNs separately in loop scheduling mode and random scheduling mode, as follows:

### 6.1. Task Prediction in Loop Scheduling

Consider that *m* of the deployed sensors on an LSN are selected to read for *s* times in a loop. Suppose that the data preparing time (Time required for a sensor to convert the monitored physical quantity into sensory data, usually recorded in the chip data sheet provided by the manufacturer. ) for the *i*-th sensor is $t_{i}$, and its control sequence and output sensory data are $c_{i}$ and $s_{i}$ bits in length, respectively. The local processing speed is *p*. If the sensory data are uploaded after every *n* sensor output is collected and merged in a packet (as described in Section 5.2), the predicted time mark ($T_{j}$) for uploading the *j*-th data package can be expressed as:

(4) $$T_{j}=\sum_{1}^{n}\left(\frac{c_{\left(i\%m\right)}+s_{\left(i\%m\right)}}{p}+t_{\left(i\%m\right)}\right)$$

In addition, at the end of the task, the LSN will upload the final data packet. If k packets have been uploaded before, the predicted time mark ($T_{end}$) for uploading the last package can be expressed as:

(5) $$T_{end}=s\sum_{1}^{m}\left(\frac{c_{i}+s_{i}}{p}+t_{i}\right)-\sum_{1}^{k}T_{j}$$

Based on Equations (4) and (5), a gateway can predict the accurate time mark for each packet uploading on an LSN and better manage tasks by estimating the future available bandwidth.

### 6.2. Task Prediction in Random Scheduling

In random scheduling mode, the data remain uploaded when *n* sensory data are collected, but each sensor may have a different proportion in the data contribution. Considering that the *i*-th sensor is read by $r_{i}$ times after the last uploading, the time mark for the next uploading (calculated from the last uploading) can be expressed as:

(6) $$T=\sum_{1}^{m}\left(\frac{c_{i}+s_{i}}{p}+t_{i}\right)*r_{i}$$

Based on the above equation, the gateway can better manage tasks by the predicted occupation and available time slots, which effectively improves the multi-task efficiency and extends the network capacity to support more LSNs.

## 7. Implementation

To verify the feasibility of our design, we implement a Butterfly prototype system and test our prototype in two proof-of-concept application scenarios.

### 7.1. Implementation Details

**Figure 10:**
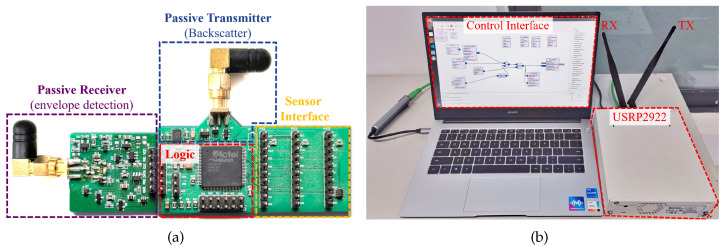
The prototype implementation of the Butterfly LSN (**a**) and the edge gateway (**b**).

The implementation of a Butterfly prototype contains two entities, i.e., a gateway and one or multiple LSNs, as follows:

- Butterfly LSN. As illustrated in Figure 10a, the logic part of the LSN prototype is implemented using an IGLOO nano FPGA (AGLN060V2), where 6.2 k of logic gates (10.3% of FPGA chip resources) are used for the logic control, and 1 kb of the memory are used for the buffer. We also deploy an ultra-low-power SIT1581 [36] oscillator to run the FPGA, which only consumes 51 μW (30 μA, 1.7 V) power at 2.5 MHz. The receiver works with an RF signal at 915 mHz with Non-Return to Zero (NZR) encoding and On-Off-Keying (OOK) modulation. We implemented the receiver by duplicating the RF envelope detection circuit in [9] but simplified the form of the transmitted signal, thus we can achieve a longer communication range. The passive transmitter generates uplink backscatter signals by controlling the on-off state of the switch MOSFET (DMG2302UK), which superimposes the sensory data on the carrier provided by the gateway. We also set three sensor interfaces on the Printed Circuit Board (PCB) to deploy different SPI-based sensors. By this, we verified the performance of Butterfly LSN by using different types of COTS sensors, including thermometers (BME280 and DS1722S), accelerometers (ADXL362, BMX160 and IIM-42351), a microphone (VM1010), and an ADC (ADS1118).
- Butterfly gateway. As presented in Figure 10b, the gateway is implemented on a software-defined radio (USRP2922) with GNU Radio software controlled by a PC, which is equipped with a 3.3 GHz i5-1035G7 CPU, 16 GB memory, and 512 GB hard disk space, running an Ubuntu 18.04 Linux operating system. The gateway can control multiple sensors on one or more LSNs wirelessly.

### 7.2. Proof-of-Concept Applications

**Figure 11:**
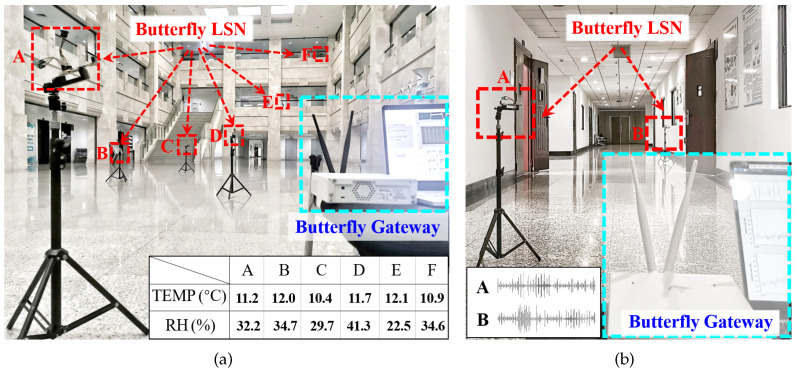
To verify the feasibility of our design, we made two proof-of-concept applications based on our prototype, i.e., an ambient air conditional meter in an open lobby (**a**) and a wireless sound collector in a corridor (**b**).

To verify the feasibility of our design, we made two proof-of-concept applications based on our prototype with different sensors as follows:

- Ambient Temperature and Humidity Meter (ATHM). As shown in Figure 11a, we monitor the air temperature and humidity in an open lobby, where six Butterfly LSNs are deployed to control the sensor for ambient sensing and return the collected sensory data to the gateway for further processing. In this application, Butterfly LSNs are equipped with a BME280 chip to collect the ambient temperature (TEMP) and Relative Humidity (RH). In the deployment of this application, all sensors deployed on all Butterfly LSNs work properly and successfully return the sensory data from six distinct locations with minimized bandwidth overhead. Specifically, to read 12,000 times of sensory data (1000 times for each sensor of the six LSN), in loop schedule mode (as described in Section 5.1), the total downlink and uplink transmission is only 1.3 kb and 47.2 kb, respectively; in real-time schedule mode, the total downlink and uplink transmission 13.3 kb and 78.9 kb, respectively.
- Wireless Sound Collector (WSC). As shown in Figure 11b, we collect the environment sound in a corridor, where two Butterfly LSNs are deployed to record ambient sound and return the collected data to the gateway. In this application, a 12-bit Analog to Digital Converter (ADC) chip (LTC1285) is deployed on the LSN to sample an analogue microphone (VM1010) for ambient sound collection and return the digitized sounds information back to the gateway. In this application, all Butterfly LSNs work properly and successfully upload the sound data from two distinct locations with minimized bandwidth overhead. Specifically, to read 30 s of sound data (150 k of samples on each LSN), in loop schedule mode (as described in Section 5.1), the total uploaded data are only 570 k bit and 513 k bit by those two LSNs, respectively (the original ADC data are 3000 k bit before de-redundancy).

## 8. Evaluation

To verify the feasibility of our design, in this section, we build up comprehensive evaluations on our Butterfly prototype in multidimensional aspects.

### 8.1. Benchmark Selection

To start with, we consider three representative nodes as the benchmark (The results of the benchmark are obtained by calculation or conversion of the results from the cited papers.), as follows:

- Benchmark 1, R2B node: the design of literature [9], an ultra-low-power node with direct radio-to-bus (R2B) communications that can directly control sensors by the gateway without the help of the general MCU. It has the simplest hardware architecture in node design that controls sensors with simple logic gates and a set of RC circuits and achieves the lowest power overhead in the state-of-the-art.
- Benchmark 2, Passive Bluetooth node: the design of literature [37], ULP sensor nodes with passive Bluetooth communications and ultra-low-power MSP430F2132 [38] MCU that can achieve data interactions with sensors down to 1.56 nJ per bit. In addition, this node has 232-bit size for each data packet, containing up to a 168-bit payload and 64-bit protocol, which can transmit multiple sensory data in one packet with good efficiency.
- Benchmark 3, simplest tradition node: we build a traditional node with the simplest design on embedded architecture as a baseline in the evaluation. It utilizes the same passive radio with our LSN design and controls sensors via an ultra-low-power STM32L431 [39] MCU. It only functions for sensor reading with the latest ULP MCU, thus eliminating all other influencing factors.

### 8.2. Task Efficiency

We first evaluate the data transmission overhead of the test nodes in sensing tasks. Given this, we deploy three sensors on each node, i.e., a 16-bit ADS1118 ADC (obtain data by sampling a VM1010 microphone), a BMX 160 gyroscope, and an ADXL 357 accelerometer.

**Figure 12:**
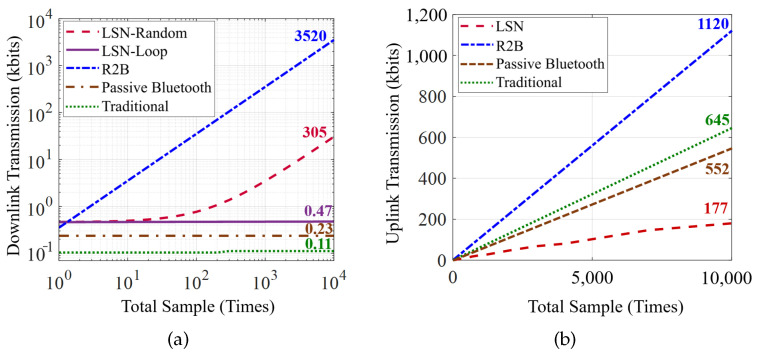
Total transmission overhead in downlink (**a**) and uplink (**b**) aspects for a Butterfly LSN compared with other three baselines in 1 to 10,000 times of sensor samples.

Figure 12 shows the transmission overhead for sampling the three sensors at different time counts, where the downlink and uplink overhead are presented in Figure 12a,b, respectively. The results show that our LSN greatly reduces the total communication overhead compared with the two baselines, especially in the LOOP scheduling mode, where only the control sequence (352 bits for these three sensors) needs to be transmitted on the downlink with simple sampling configuration, i.e., sampling rate and time duration. In contrast, the random schedule mode takes slightly more as the schedule sequence with 1 bit per sensor decision (0 for skip and 1 for read, as described in Section 5.1) needs to be added. Although the passive Bluetooth node and traditional node achieve lower downlink overhead as it has a built-in embedded program for sensor control that only needs to transmit one command to start, and the LSN wins a lot in the uplink with the last bit transmission mechanism (as described in Section 5.2). By contrast, the R2B node takes the highest overhead as all sensors deployed on it are directly controlled by the gateway, where each sampling requires the full transmission of the control sequence and raw sensory data in the downlink and uplink, respectively.

**Figure 13:**
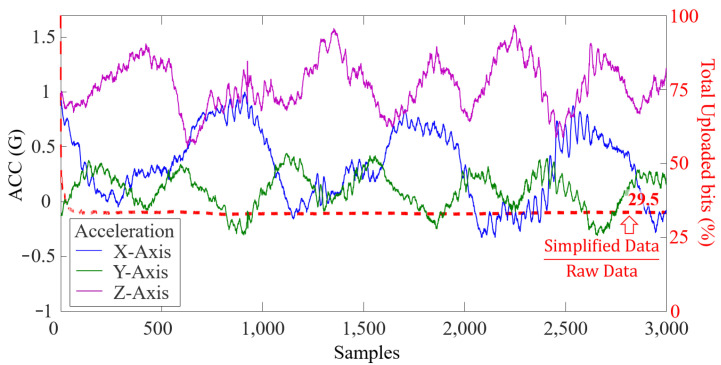
The design of LSN reduces 70.5% of uplink loads when uploading 3000 samples of the data from a three-axis accelerometer.

To better present the effect of our last bit transmission mechanism, we intercepted the sampling result of a piece of accelerometer data, as shown in Figure 13. It can be seen that the mechanism reduces 70.5% of uplink overhead where the transmitted data are only 29.5% of the raw data after removing redundancy.

### 8.3. Extreme Performance

**Figure 14:**
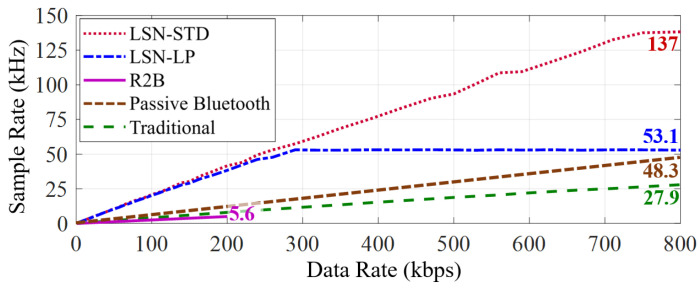
Our LSN achieves a much higher sample rate for the three sensors mentioned compared with the three selected benchmarks.

Next, we deploy a 12 bit high-speed ADC (The high-speed ADC we selected is only a tool for evaluations that seek the maximum task rate for tested nodes. It is not a part of our prototype, so we do not consider its power consumption for the test results.) (MCP33141-05), which can be continuously sampled at up to 500 kHz, thus eliminating the impact of sensor data conversion delays on the node’s performance. We utilize a signal generator (AFG1062) to generate analog signals for the ADC sampling. As only one ADC is deployed, the LSN work in Loop scheduling mode and the data are uploaded per 64 samplings and merged in a packet after simplifying (Section 5.2). We made two versions of LSN for the test, i.e., a standard (STD) version drive with 2.5 MHz clock and a low-power (LP) version drive with a 1 MHz clock. The clock is all provided by the SIT 1582 series crystal oscillators where the 2.5 MHz version consumes 51 μW (30 μA, 1.7 V) and the 1 MHz version consumes 22 μW (13 μA, 1.7 V). The results are shown in Figure 14.

It can be seen that the Butterfly LSN has the best performance compared with the three selected baselines. Benefiting from the design of DESC (as described in Section 5), the average communication overhead of each sample on LSN is only 4.7 bits, containing the cost of data packing. In addition, with the design of EBST (as described in Section 4), our Butterfly LSN achieves four times the maximum data transmission rate (800 kbps) than the R2B node (200 kbps) while using the same low-power envelope detector circuit. The limitation of LSN is the speed of local clock, where the standard (STD) and low power (LP) versions reach the sampling rate of 137 kHz and 53.1kHz at data rates of 290 kbps and 750 kbps, respectively. To further accurate the task rate, a local clock with a higher frequency could be useful, but it brings additional power overhead accordingly.

In contrast, although the passive Bluetooth node and traditional node can control sensors via its built-in embedded program that requires less downlink control, the raw sensory data uplink also becomes a bottleneck of the achieved sampling rate. Among them, the passive Bluetooth node can upload 14 samples data in one packet (with 168 bits of the payload) and has the best performance in the selected benchmarks, but our LSN can also achieve a 2.83 times higher sample rate than it. In contrast, the R2B node achieves the lowest sampling rate due to the mass data transmission in every sample, i.e., 36 bit containing raw sampling data, control sequence and protocol. Ultimately, compared to the selected three benchmarks, i.e., R2B, passive Bluetooth, and traditional nodes, our LSN achieves 24.5, 2.83, and 4.91 times the maximum sample rates, respectively.

### 8.4. Power Overhead

To estimate the power consumption of our LSN, we utilize Libero SoC v12.0 [40] software for simulation. We write Verilog codes and use the default configuration for the evaluation, where the results are presented in Table 1. We can find that the main power consumption comes from the functions of Data-Efficient Sensor Control (DESC, as described in Section 5), where 5.4 k (9% of FPGA chip resources) of logic gates are used in this part. In contrast, the overhead of Efficient Bus Signal Transmission (EBST, as described in Section 4) and static power of the FPGA chip are relatively low, as the EBST functions only take 0.8 k of logic gates (1.3% of FPGA chip resources), and the FPGA chip only takes 11 μW in static. The rest part of the node includes a crystal oscillator, an envelope detection circuit, and a switching MOSFET for backscattering. Since the envelope detection is mainly driven by RF power and the MOSFET switch has high impedance in its gate, it consumes current. Hence, the power consumption of the rest part mainly comes from the 2.5 MHz crystal oscillator. Based on such design, an LSN can sample the ADC at 137.3 kHz with only 703 μW power overhead, thus only needing 5.12 nJ power to sample the ADC once.

**Figure 15:**
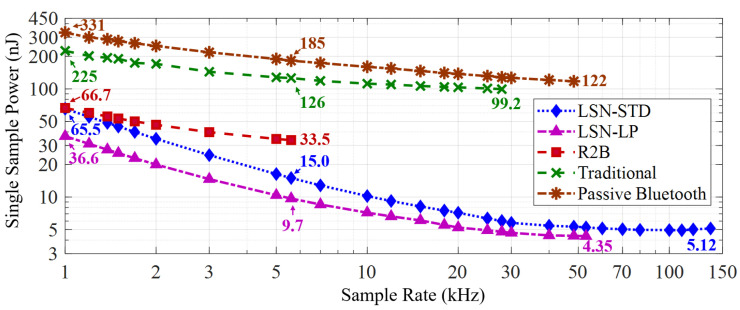
The Butterfly LSN has down to 4.35 nJ power overhead to sample a sensor once, which is far from that of R2B nodes (33.5 nJ), passive Bluetooth nodes (122 nJ) and traditional nodes (99.2 nJ).

Next, we compared our LSN with the three selected baselines on the single sampling overhead. For the baseline, the data of R2B nodes and passive Bluetooth are calculated from [9] their literature [9,37], and the data of traditional nodes are estimated (To ensure the fairness of the comparison, the MCU’s power overhead was obtained from its official documentation, where we tested its required running speed and found corresponding value for the evaluation) based on its low-power description file [41]. The results are demonstrated in Figure 15.

The results demonstrate that both the standard (STD) and low-power (LP) version have much less power consumption in a single ADC sample compared with the selected three baselines. In addition, with the increase of sampling rates, the static overhead on LSNs are shared evenly, thus effectively reducing the power consumption on each sampling. For the standard version, it can sample the 12-bit ADC at 137.3 kHz with only 703 μW overhead, thus only consuming 5.12 nJ power for each sample. For the low-power version, the power overhead of a single sample is down to 4.35 nJ. In contrast, the passive Bluetooth and traditional nodes have higher power consumption due to the overhead of MCUs. Despite the passive Bluetooth nodes only requiring 1.56 nJ per bit (25.9 nJ for a single task) for data transmission, the MCU brings major power overheads in mWs to reach high task rates. In addition, although R2B has the simplest architecture for sensor control and achieves the lowest power consumption in the state-of-the-art, its overhead on a single task is still much higher than that of LSN due to mass data transmission. As a result, compared with the R2B, passive Bluetooth, and traditional nodes, the standard LSN can save up to 84.7%, 95.8%, and 94.3% power overhead to sample a sensor once, respectively. For LSNs in the low-power version, the saved power becomes 87%, 96.4%, and 95.6% compared to the R2B, passive Bluetooth and traditional nodes, respectively—satisfying both the requirements of high task rate and low power overhead simultaneously.

### 8.5. Effectiveness of the Task Prediction Mechanism

**Figure 16:**
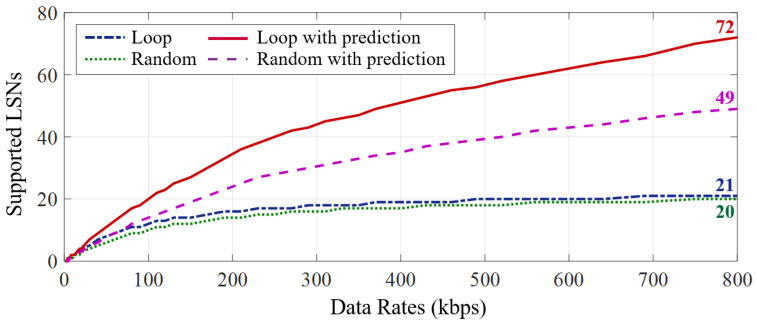
The task prediction mechanism can significantly improve bandwidth utilization efficiency in both the loop and random scheduling mode, which significantly increases the gateway network capacity that can satisfy the data transmission requirements for more LSNs.

We evaluate the effect of our task prediction mechanism through simulation, where three sensors are virtualized to deploy on each LSN, i.e., a 3-axis accelerometer, a 16 bit ADC, and a gyroscope, whose conversion delay is set to 25 μs, 10 μs and 60 μs, respectively. The simulation is established on Matlab 2021a, where the monitored physical quantity varies randomly within the range of the sensor, and all sensors on all LSNs are sampled at 200 Hz. We configure all simulated LSNs the same as the standard version, which process data at 2.5 MHz (as described in Section 8.3). In loop reading mode, each LSN is set to reads above sensors for 200 cycles; in random reading mode, the gateway transmits the reading sequence for an LSN in every 30 sensor readings. The results of supported devices under different gateway communication data rates are presented in Figure 16.

The results demonstrate that the task prediction mechanism significantly improves the network capacity by 3.42 and 2.45 times in the loop schedule and random schedule mode, respectively. In the absence of the task prediction mechanism, the gateway can only perform the tasks on each LSN serially, which brings a lot of waiting time for uploading sensor data processed by the LSN, resulting in a huge waste of bandwidth. In contrast, benefiting from the task prediction mechanism, tasks on multiple LSNs can be parallel, thus significantly reducing the waiting cost and improving the network capacity that can support data transmission for more LSNs.

### 8.6. System Coverage

**Figure 17:**
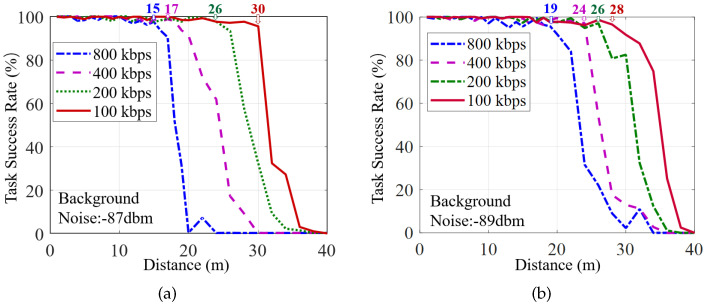
The task success rate of Butterfly LSN in different distances from the gateway tested in an open lobby (**a**) and a corridor with rich multi-paths (**b**).

While the LSN utilizes backscatter communication for ultra-low-power, the coverage evaluations become essential where package loss may result in task failure if the LSN is too far from the gateway. Hence, we evaluate the task execution success rate of nodes at different distances for each of the two scenarios mentioned in Section 7.2, where task success is defined as a complete data package that is successfully returned to the gateway; otherwise, it is a failure. The gateway transmits the signal at 30 dBm via a Low Noise Amplifier (LNA) at 915 MHz to the LSN for sensor reading. The results are presented in Figure 17.

The results demonstrate that, at 800 kbps data rates, an LSN can be deployed at 17 m and 19 m to the gateway in the lobby and corridor scenarios, respectively, where almost all (≥95%) tasks can be successfully performed. In addition, the coverage of the gateway can be further improved by reducing the data rate appropriately. For 400 kbps, the communication distance from the gateway to the LSN can reach 17 m and 24 m in the lobby and corridor scenarios, respectively. For 100 kbps, the coverage distance can be increased to 30 m and 28 m in these two scenarios, respectively. Since the corridor has lower background noise, and the multi-path can enhance the transmitted signal, a Butterfly system can achieve greater coverage in the corridor than that in the lobby.

We can also see that the task success rate has a sudden drop when the distance reaches a certain value in both of the two scenarios above. The reason stems from the fact that the LSN utilizes the envelope detection circuit to receive signals passively. When the signal strength drops to the threshold, it works unstably. As a result, the Butterfly prototype can be well applied to indoor scenes (within 30 m), such as smart homes [42,43,44]. For outdoor or long-distance required applications such as smart factories [45] and smart cities [46], active communications (e.g., Lora [47], NB-IoT [48]) with longer communication ranges are more suitable to be deployed, which is also our future work.

## 9. Discussion

In this paper, we settle three key challenges that obstruct our design. Nevertheless, we believe that two additional points should be discussed to instruct our further research.

(1) Security. The removal of local MCU enhances the security of LSNs, i.e., attackers cannot obtain data by stealing the node as it contains no embedded program that avoids the security risks with key program data loss. Even if the buffered data are stolen by technical attackers, it is mostly useless for them as the data are designed for the control of COTS sensors and thus contain no privacy information. To further enhance the security of our Butterfly system, the data transmission between the gateway and LSNs could be a key consideration. Specifically, to enhance system security, lightweight encryption schemes like PRESENT [49], Data Encryption Standard Lightweight (DESL) [50], and Light Encryption Device (LED) [51] are possible to be incorporated into our further design with FSM logic design with no more than 2K logic gates (3.3% resources on the AGLN060 FPGA chip). In addition, we would also establish a whitelist mechanism on the gateway to ensure the legitimacy of nodes in the network by storing the information (device ID, sensor data structure, etc.) of legitimate nodes. Moreover, it is also possible for us to utilize hardware Trojan detecting methods [52,53,54,55] and design authentication mechanisms for sensor nodes to prevent possible attacks caused by wireless Trojan nodes connected by malicious users.
(2) Error detection and data correction. In this paper, we realized the basic idea of Butterfly and achieved both ultra-low-power and high task throughput on node design. In future designs, we consider that the error detection and correction in node communication links is a key consideration that ensures the system robustness in practical applications. Specifically, we might incorporate bit level error detection and correction methods like Cyclic Redundancy Checksum (CRC) [56,57,58] and MD5 Message-Digest Algorithm (MD5) [59] by hardware FSM design with logic circuits to give the node a robust communication link. In addition, it is also possible to design the hardware FSM with logic gates to solidify functions of intelligent data recovery [60,61,62] and re-transmission [63] to further optimize the communication robustness in environments that has strong signal interference.
(3) ASIC-based local processing. To further optimize the communication overhead and reduce power consumption, we consider localizing commonly used calculations with a simple ASIC design to further reduce requirements of sensory data transmission in some scenarios. For instance, in temperature monitoring scenarios, if the application only needs an alarm signal for over-threshold temperature notification, which does not need to upload all the sensed data and processing on the gateway. In that scenario, the sensed temperature data should be compared with the preset threshold value locally on the node, and the alarm information could only be uploaded when the sensed value touches the threshold. To achieve this, we consider curing some common computational logic (e.g., numeric value comparison, data averaging) with ASIC design and implementation in the future, which might further reduce the node’s power consumption while improving its task performance simultaneously.

## 10. Conclusions

In this paper, we propose Butterfly, a methodology of system design on both node architecture and gateway functions to support μW level ULP sensor nodes with rapid task performance. To this end, we offload all application-related tasks from each LSN to the edge gateway, thus minimizing task loads and power overheads on LSNs by only keeping the bottommost functions of the sensor reading. To boost task rates, we design efficient bus signal transmission and data utilization mechanisms on the LSN to achieve rapid task execution within a given communication data rate. We also set up a task prediction mechanism on the gateway to predict the task progress on each LSN to better manage tasks when they need to be executed concurrently. We implement a Butterfly prototype and make comprehensive evaluations with two proof-of-concept applications. The results show that a Butterfly LSN can sample a 12-bit ADC at 137.3 kHz at only 703 μW overhead, which only needs 5.12 nJ power for each sample. Compared with traditional ULP sensor nodes, the LSN can speed up the task rate by 4.91 times while reducing the power consumption by 94.3% in processing the same task, respectively. In addition, Butterfly nodes have natural security advantages (e.g., anti-capture) benefited by the fact that they offload all application-related functions to the gateway and contain no private data in local, thus resistance to attackers.

## Figures and Tables

**Table 1:** Power breakdown and task performance of Butterfly LSNs (STD version) in different communication data rates.

Data Rate	FSM			Rest ^4^ Part	Total	Sample Rate	Single Sample
	EBST ^1^	DESC ^2^	Static ^3^				
50 kbps	7 μW	34 μW	11 μW	57 μW	109 μW	10.8 kHz	10.1 nJ
100 kbps	13 μW	67 μW	11 μW	58 μW	149 μW	21.5 kHz	6.93 nJ
200 kbps	22 μW	132 μW	11 μW	60 μW	225 μW	42.7 khz	5.27 nJ
400 kbps	43 μW	261 μW	11 μW	63 μW	378 μW	75.2 kHz	5.03 nJ
600 kbps	65 μW	397 μW	11 μW	68 μW	541 μW	109.8 kHz	4.93 nJ
800 kbps	86 μW	532 μW	11 μW	74 μW	703 μW	137.3 kHz	5.12 nJ

^1^ The function of Effective Bus Signal Transmission, the design details are presented in Section 4. ^2^ The function of Data-Efficient Sensor Control, the design details are presented in Section 5. ^3^ The static power of the AGLN060 FPGA chip, which requires 8 μA at 1.4 V. ^4^ The rest part of an LSN, which contains a 2.5 MHz crystal clock to run the FPGA, an envelope detection circuit for downlink receiving and a MOSFET switch for backscatter uploading.

## Data Availability

The data presented in this study are available on request from the first author.

## Abbreviations

The following abbreviations are used in this manuscript:

IoT	Internet of Things
SPI	Serial Peripheral Interface
COTS	Commercial-Off-the-shelf
ULP	Ultra-Low-Power
MCU	Microcontroller
LSN	Lightweight Sensor Node
FSM	Finite State Machine
EBST	Efficient Bus Signal Transmission
DESC	Data-Efficient Sensor Control
TPP	Task Progress Prediction
